# Optimizing the Conditions for Whole-Genome Sequencing of Avian Reoviruses

**DOI:** 10.3390/v15091938

**Published:** 2023-09-16

**Authors:** Sonsiray Alvarez Narvaez, Telvin L. Harrell, Olatunde Oluwayinka, Holly S. Sellers, Zubair Khalid, Ruediger Hauck, Erfan U. Chowdhury, Steven J. Conrad

**Affiliations:** 1 US Department of Agriculture, Agricultural Research Service, Southeast Poultry Research Laboratory, Athens, GA 30605, USA; sonsiray.alvarezarvaez@usda.gov (S.A.N.); telvin.harrell@usda.gov (T.L.H.); olatunde.oluwayinka@usda.gov (O.O.); 2 Poultry Diagnostic and Research Center, Department of Population Health, College of Veterinary Medicine, University of Georgia, Athens, GA 30602, USA; hsellers@uga.edu; 3 Department of Pathobiology, College of Veterinary Medicine, Auburn University, Auburn, AL 36849, USA; zzk0012@auburn.edu (Z.K.); mrh0079@auburn.edu (R.H.); 4 Department of Poultry Science, College of Agriculture, Auburn University, Auburn, AL 36849, USA; 5 Alabama Department of Agriculture and Industries, Veterinary Diagnostic Laboratory System, Auburn, AL 36832, USA; euc0001@auburn.edu

**Keywords:** avian reovirus (ARV), whole-genome sequencing (WGS), next-generation sequencing (NGS), virion purification, virion enrichment, ribosomal RNA depletion

## Abstract

Whole-genome sequencing (WGS) is becoming an essential tool to characterize the genomes of avian reovirus (ARV), a viral disease of economic significance to poultry producers. The current strategies and procedures used to obtain the complete genome sequences of ARV isolates are not cost-effective because most of the genetic material data resulting from next-generation sequencing belong to the host and cannot be used to assemble the viral genome. The purpose of this study was to develop a workflow to enrich the ARV genomic content in a sample before subjecting it to next-generation sequencing (NGS). Herein, we compare four different ARV purification and enrichment approaches at the virion, RNA and cDNA levels to determine which treatment or treatment combination would provide a higher proportion of ARV-specific reads after WGS. Seven ARV isolates were subjected to different combinations of virion purification via ultracentrifugation in sucrose density gradient or Capto Core 700 resin with or without a subsequent Benzonase treatment, followed by a chicken rRNA depletion step after RNA extraction and a final ARV cDNA amplification step using a single-primer amplification assay. Our results show that the combination of Capto Core 700 resin, Chicken rRNA depletion and cDNA amplification is the most cost-effective strategy to obtain ARV whole genomes after short-read sequencing.

## 1. Introduction

Avian orthoreoviruses (avian reoviruses, ARVs) are a persistent challenge to poultry producers in the United States and globally. Infection with ARVs has been associated with a variety of symptoms and syndromes in commercial poultry, including tenosynovitis/viral arthritis, enteric symptoms such as watery diarrhea, respiratory symptoms, myocarditis, viral hepatitis, runting-stunting syndrome (RSS), poor feed conversion with compromised weight gain and high rates of morbidity, and occasionally, mortality [1,2,3,4]. ARVs are ubiquitous among poultry flocks, and despite vaccination efforts, the frequency of ARV outbreaks affecting broiler chickens in the United States, Canada and China has increased in the last decade [2,3,5,6]. ARVs belong to the *Orthoreovirus* genus of the *Reoviridae* family, and their genome consists of ten double-stranded RNA segments classified into three large (L1, L2 and L3), three medium (M1, M2 and M3) and four small (S1, S2, S3 and S4) segments with sizes that vary between 1100 and 4000 bp [7]. Traditionally, ARV genomic classification into genogroups has been based exclusively upon a single gene encoded by the S1 chromosomal segment, the SigmaC gene. The SigmaC (σC) gene product is the primary antigenic viral protein to which neutralizing antibodies bind and is the best characterized gene and gene product of the ARVs [2,4,8,9]. However, there is not a strong correlation between the SigmaC sequence (or genogroups based upon SigmaC sequence) and the observed pathogenicity and clinical presentation of the strain in the field [9].

With the arrival of next-generation sequencing (NGS) technology, whole-genome sequencing (WGS) has become an important tool to determine the genetic background of ARV strains that escape neutralizing vaccine responses [2,10]. Although the workflow for the production of NGS data appears to be straightforward, the NGS of dsRNA viral genomes introduces some complications and is a relatively new practice for which “gold standards” and widely-accepted best practices have not yet been established. Viruses are obligated intracellular pathogens. If no enrichment of the viral genetic material or depletion of host genetic material is done, the majority of the reads recovered will map to the host genome, while only a very small percentage will correspond to the targeted virus [2]. Even by disregarding the economic losses associated with up to >90% of the sequencing cartridge being occupied by non-target sequences, without a highly pure viral genomic sample, the sequencing run may not yield enough targeted reads to have sufficiently complete genome coverage, and therefore, a reliable genome assembly [11].

Currently, there are several publications that report the use of WGS to determine the genetic relatedness of ARV strains isolated from poultry, most of which use short-read sequencing technology and different enrichment strategies to overcome host genomic contamination. Generally researchers opt for sequencing a small number of ARV isolates per sequencing run, hoping to obtain enough viral reads for good coverage [12]. Egaña-Labrin and collaborators introduced a host rRNA depletion step with a Terminator^TM^ 5′-Phosphate-Dependent Exonuclease degradation step (the degradation of RNAs without a 5′ triphosphate cap) before converting the ARV RNA genome into cDNA [2], while Chrzastek et al. [13] used a reovirus single primer amplification (R-SPA) approach to enrich ARV cDNA. There are no reports showing if these intermediate steps significantly reduced host contamination and/or enriched for ARV-mapping reads after sequencing. James and colleagues first developed a protocol for the purification of ARV virions from crude cell lysates [14] using Capto Core 700 kDa resin, which we have integrated into our purification/isolation scheme.

**Figure 1:**
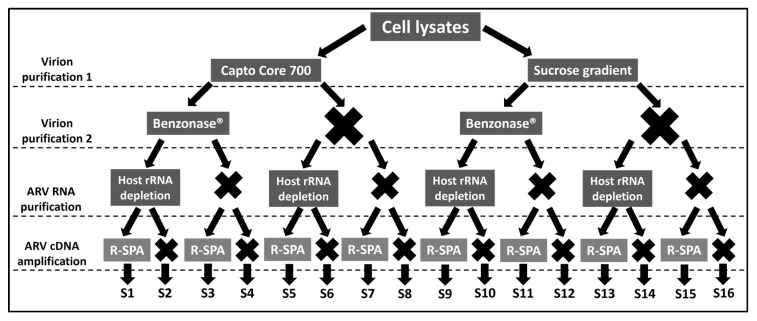
Schematic representation of the overall research strategy followed in this project, in which sixteen different combinations (S1–S16) of four ARV purification/amplification methods (left) were tested.

Herein, we have performed a systematic comparison of four different approaches (see Figure 1) for ARV purification and enrichment at different levels (virion, RNA and cDNA) to determine which workflow will generate the higher number of ARV-reads after NGS, with the aim of setting up a gold standard for ARV WGS. Our protocol has been entered into protocols.io (DOI: dx.doi.org/10.17504/protocols.io.14egn38z6l5d/v1, accessed on 29 June 2023) and is available to the public.

## 2. Materials and Methods

### 2.1. Experimental Design

This section details the overall experimental procedure, while the subsections (below) provide details for each procedure. Sixteen different combinations involving four purification steps were tested in these experiments, each of which is detailed in Figure 1. Briefly, five T175 flasks of LMH cell (ATCC CRL-2117) monolayers at 95% confluency were infected with approximately 20 μL of ARV-infected cell culture supernatant and placed in an incubator (38 °C, humidified, with 5% CO_2_) for 5 days. After that time, the infected LMH cells and supernatant were harvested, centrifuged at 3000× *g* for 10 min at room temperature (RT), and the pellet was resuspended in 1600 µL of Virus Dilution Buffer (VDB) [14]. Cells were lysed via sonication on ice (3 pulses at 30% amplitude, 10 s on and 30 s off, using a Branson Digital Sonifier 450 (Branson Ultrasonics Corporation, Brookfield, CT, USA). The initial 1600 µL of infected cell lysate was split into two aliquots of 800 µL each that were subjected to virion enrichment using Capto Core 700 resin (Cytiva, Marlborough, MA, USA, catalog number GE17-5481-01), or ultracentrifugation on a sucrose gradient. This resulted in two vials of enriched virions at a volume of 375 µL each. Then, each of the vials were divided into two, and 187.5 µL of each vial (~1.25 T175 flask of infected cells) was treated with the nuclease Benzonase (Sigma-Aldrich, St. Louis, Missouri, catalogue number 9025-65-4), while the other 187.5 µL remained untreated. After this second purification step, there were four 187.5 µL vials containing purified virions (two of which were treated with Benzonase, and two were untreated) from which RNA was extracted. Once again, each of the four virion preparations was divided into two, and half of each extract was subjected to host ribosomal RNA (rRNA) depletion, while the other half remained untreated. After this third purification step, there were eight vials of RNA (four whose rRNA was depleted, and four were untreated) that were converted to cDNA via reverse transcription. Finally, each of the eight cDNA samples were split into two, and half of the sample received ARV genome amplification using the R-SPA method, and the other half did not. The sixteen DNA samples resulting from this experiment were sequenced in a single short-read sequencing run.

For subsequent experiments in which the number of treatment combinations was reduced from sixteen to eight or to four, we reduced the number of infected cells (for testing eight treatment conditions, two T175 flasks were used, and one T175 flask was used when testing four) and volumes for purification steps one and two, accordingly. In total, seven ARV strains/isolates were used in this study (Table S1).

### 2.2. Cell Lines and Culture Conditions

The LMH (chicken epithelial liver from ATCC, catalog number CRL-2117) cell line was propagated in Dulbecco’s Modified Eagle Medium (DMEM, Gibco, Waltham, MA, USA, catalog number 1195-065) supplemented with 8% heat-inactivated fetal bovine serum (FBS, R&D Systems, catalog number S11150) and 0.1% gentamycin antibiotic (Lonza, Walkerville, MD, USA, catalog number 17-518L). The cells were maintained at 38 °C and 5% CO_2_ with humidification.

### 2.3. Purification of ARV Virions Using Capto Core 700 Resin

ARV virion preparations were purified using the Capto700 slurry protocol previously described by James et al. [14]. Briefly, 350 µL of infected lysed cells in VDB were centrifuged at 800× *g* for 10 min to remove the nuclei and cell debris. The supernatant was collected in a fresh tube containing 100 µL of 50% Capto700 slurry. The samples were mixed in an end-over-end tumbler for 45 min at room temperature and subsequently centrifuged at 800× *g* for 10 min. The top phase was transferred to a new tube containing 100 µL of 50% Capto700 slurry for another purification round in the end-over-end tumbler (45 min at room temperature). To clear all the Capto Core 700 resin, the samples were passed through an Illustra MicroSpin column (GE Healthcare, catalog number GE27-3565-01) at 800× *g* for 5 min. Viruses were kept at 4 °C until processed for RNA.

### 2.4. Purification of ARV Virions Using a Sucrose Gradient

LMH-ARV crude lysate was made via the ultracentrifugation of cell culture media and debris at 32,000 rpm for 2 h at 4 °C. The LMH-ARV crude pellet was resuspended in 800μL DMEM (supplemented as described above) after centrifugation and layered on a 4-step sucrose density gradient. The sucrose density gradient was prepared from a stock 3 M sucrose working solution that was further diluted with nuclease-free H_2_O at 48%, 30%, and 15% of the original stock. The following ratios of sucrose and H_2_O from the bottom to top layers were as follows: 700 μL 3 M sucrose (1.59 g/cm^3^); 494 μL and 205 μL (48%) (1.23 g/cm^3^); 308 μL and 391 μL (30%) (1.13 g/g/cm^3^); and 154 μL and 545 μL (15%) (1.05 g/cm^3^), respectively. The crude lysate was layered gently over the gradient and centrifuged in an Optima L- 90 K ultracentrifuge (Beckman Coulter, Brea, CA, USA) at 36,000 rpm using a SW60 rotor for 4 hrs at 4 °C. The lower band of the gradient (enriched virions) was collected and resuspended in a 30% sucrose solution. The resulting suspension was ultracentrifuged again for 2 h under the same conditions (36,000 rpm at 4 °C in a SW60 rotor (Beckman Coulter, catalogue number 335650) to pellet the virions out of the sucrose solution. Finally, the virion pellet was resuspended in nuclease buffer (see below) for downstream procedures.

### 2.5. Nuclease Treatment

Purified virions were subjected to treatment with Benzonase. Purified virions from 2.5 T175 flasks of cells were pelleted (36,000 rpm for 4 h at 4 °C) and resuspended in 187.5 µL nuclease buffer (10 mM Tris HCl pH 7.5; 2 mM MgCl_2_; 10% sucrose) and mixed with 2.25 µL of Benzonase (~562 U) and incubated at 37 °C for 30 min. The reaction was stopped with 9 µL of 0.5 M EDTA.

### 2.6. RNA Extraction and Host/Bacteria rRNA Depletion

RNA was extracted using MagMAX™ Viral RNA Isolation Kit (Applied Biosystems, Waltham, MA, USA, catalog number AM1939) from an initial volume of 350 µL following the manufacturer’s protocol. Bacterial and host rRNA depletion was performed using the protocol from Parris et al. [15]. Briefly, 1 µL of ssDNA probes (10 µM, IDT) was hybridized with 12 µL of total RNA in the presence of 2 µL NEB Probe Hybridization Buffer (New England Biolabs, Ipswich, MA, USA). Hybridization was conducted in a thermal cycler (BioRad, Hercules, CA, USA, model T100) at 95 °C for 2 min, followed by a decreasing temperature cycle from 95 °C to 22 °C at 0.1 °C/second and a final incubation at 22 °C for 5 min. The probe-bound rRNAs present in the sample were degraded immediately after hybridization with 2 µL of RNase H enzyme (New England Biolabs, catalogue number M0297S), 2 µL RNase H reaction buffer, and 1 µL of nuclease-free water with 5 µL of the hybridization mix (incubation at 37 °C for 30 min). Finally, the unbound ssDNA probes were digested at 37 °C for 30 min from each sample (10 µL) using 2.5 µL DNase I enzyme (New England Biolabs, catalog number M0303S) in the presence of DNase I reaction buffer (5 µL) and nuclease-free water (22.5 µL). The resulting rRNA-depleted RNA sample was further purified using 2.2X RNAClean XP beads (Beckman Coulter, catalog number A63987) following the manufacturer’s recommended protocol. RNA concentrations and the percentage of rRNA contamination were measured with a 2100 Bioanalyzer system (Agilent, Santa Clara, CA, USA) and the RNA 6000 Pico Kit (Agilent, catalog number 5067-1513).

### 2.7. cDNA Production and Amplification using R-SPA

The single primer amplification protocol (R-SPA) described by Chrzastek et al. [13] was used to produce ARV cDNA for whole-genome sequencing. Briefly, SuperScript IV reverse transcriptase (ThermoFisher Scientific, Waltham, MA, USA, catalog number 18090010) was used to convert ARV RNA into cDNA. First, 10 µL of RNA was mixed with 1 µL of 100 µM primer R8N (produced using our specifications by Integrated DNA Technologies), 1 µL of 10 mM dNTP mix (New England BioLabs, catalog number N0447S), and 1 µL of nuclease free water and incubated at 95 °C for 4 min, followed by it being left for 1 min on ice. The mix was then added to 7 µL of SuperScript IV reaction mix consisting of 4 µL of 5X buffer, 1 µL of 100 µM DTT (ThermoFisher Scientific, included in the SuperScript IV mix), 1 uL recombinant RNaseOUT Recombinant Ribonuclease Inhibitor (40 U/μL, ThermoFisher Scientific, catalog number 10777019) and 1 μL of SuperScript IV reverse transcriptase (200 U/ μL). The 20 μL reaction was incubated in a T100 Thermal Cycler (BioRad) under the following conditions: 10 min at 23 °C, and 10 min at 55 °C, followed by 10 min at 80 °C. cDNA second strand synthesis was performed immediately using Klenow polymerase (New England Biolabs, product number M0210S). First, 1 μL of primer 10 μM R8N (Integrated DNA Technologies (IDT)), 1 µL of 10 mM dNTP mix (New England BioLabs, catalog number N0447S) and 2 µL of 10X Reaction Buffer were added to the 20 µL of the previous step, and the mix was incubated at 94 °C for 3 min and cooled to 4 °C in a thermocycler. Then, 1 uL of Klenow polymerase was added to the reaction (final volume 25 uL), and the mix was incubated at 37 °C for 60 min. cDNA was purified using X1.8 AMPure XP beads (Beckman Coulter, product number A63880) following the manufacturer’s protocol and subsequently amplified using the HiFi PCR Phusion kit (New England Biolabs, catalog number M0530S). The PCR reaction mix conditions were as follows: 31 μL of nuclease free water, 10 μL of 1X Phusion HF buffer, 1 μL of 10 mM dNTP mix, 2.5 μL of primer R (10 μM) and 0.5μL of Phusion DNA polymerase. The 45 μL mix was incubated in a thermal cycler (BioRad, model T100) under the following conditions: 30 s of denaturation at 98 °C; 35 amplification cycles of 30 s at 98 °C; 30 s at 50 °C; 1 min at 72 °C; and a final 10 min extension at 72 °C. The PCR products were again purified using X1.8 AMPure XP beads following the manufacturer’s protocol.

### 2.8. Whole-Genome Sequencing and Bioinformatic Analysis

Genomic libraries were generated with the Nextera XT DNA Library Preparation Kit (Illumina, San Diego, CA, USA catalog number FC-131-1024) and IDT for Illumina DNA/RNA UD Indexes Set A (Illumina, catalog number 20027213). Samples were run using a MiSeq Reagent Nano Kit v2 500 cycles cartridge (Illumina, catalog number MS-103-1003) on an Illumina Miseq instrument (Illumina). Raw reads were trimmed, and quality-filtered (Phred score > 30) using Trimmomatic [16]. High-quality reads were mapped against the chicken genome (NCBI accession no. GCF_016699485.2) using BWA aligner [17]. Reads that did not map against the chicken genome (potential ARV reads) were extracted using SAMtools v1.16.1 [18] and used for de novo assembly using SPAdes v3.15.3 [19] and reference-guided assembly with MIRA v3.4 in GalaxyTrakr [20]. The generated contigs were mapped to the ARV strain S1133 genome (NCBI accession number KF741756–KF741765) and extracted using Geneious mapper with the highest sensitivity and set to five iterations (Geneious Prime 2022.1.1, https://www.geneious.com). ARV-mapping contigs were used to produce complete genomes that were polished using Pilon v1.24 [21]. The quality of the assemblies and estimated genome length was assessed using QUAST [22] Galaxy v5.2.0+galaxy1 was also used on extracted ARV-mapping contigs in GalaxyTrakr [20]. Genome coverage was estimated using SAMtools after mapping the filtered reads (with BWA) to each isolate’s complete genome.

### 2.9. Statistical Analysis

For the data generated in the first sequencing run, two-way ANOVA with Tukey’s multiple comparisons post hoc test was performed to elucidate potential significant differences in the number of raw reads, quality-filtered reads and ARV-mapping reads between the samples that went under virion purification using Capto Core 700 resin and sucrose gradient with and without subsequent R-SPA amplification. For the analysis of subsequent experiments, one-way ANOVA with Tukey’s multiple comparisons post hoc test was performed to elucidate potential significant differences in the number of raw reads, quality-filtered reads, proportion of chicken genome-mapping reads, proportion of ARV-mapping reads, number of contigs and ARV-mapping contigs between the samples that were subjected to different purification methods. All statistical analysis were carried out using statistical software GraphPad Prism version 9.3.1 (GraphPad Software, San Diego, CA, USA).

## 3. Results

### 3.1. Single Primer Amplification of ARV cDNA (R-SPA) Significantly Increases the Number of ARV-Mapping Reads Recovered after WGS

The clinical ARV isolate “Alabama” was plaque-purified (three rounds), subsequently expanded in five T175 flasks of LMH cells at SEPRL, and subjected to sixteen different combinations of four purification steps (Figure 1). Hence, sixteen samples were included in the first sequencing run that yielded a total of 1,067,010 raw (66,688 ± 17,229 (Mean ± SEM)) reads, from which 721,698 (45,106 ± 13,758 (Mean ± SEM)) passed the quality filter. Marked differences in the number of raw reads, filtered reads and mapping reads were observed between the two virion purification methods (Capto Core 700 resin and sucrose gradient), as well as between the samples that received ARV-cDNA amplification and the samples that did not (Table 1). The four samples (S9–S12) in which virions were purified with the sucrose gradient, but did not receive R-SPA, resulted in a smaller number of raw reads than those of the other groups (even when the genomic libraires were normalized prior to pooling and sequencing) and the lowest proportion of quality-filtered reads. Only one of the samples of this group (S10) had enough non-host filtered reads to produce an assembly. With regard to the number of mapping reads, neither of the virion purification methods (Capto Core 700 resin or sucrose gradient) were efficient themselves (samples S1, S5, S9 and S13) at reducing the number of reads mapping to the chicken genome, which exceeded 75% of all the reads (Table 1). Benzonase treatment (samples S2, S6, S10 and S14) decreased the number of chicken-mapping reads and increased the number of ARV-mapping reads regardless of the virion purification method used. This was not the case for the host rRNA depletion step (samples S3, S7, S11 and S15) that only showed a difference when combined with Capto Core 700 for virion purification. The treatment combination that yielded the highest proportion of ARV-mapping reads (77.6%) was virion enrichment via the sucrose gradient followed by a Benzonase treatment and R-SPA cDNA amplification (sample S14, Table 1).

Overall, the samples whose ARV-cDNAs were amplified with R-SPA showed a consistently higher percentage of ARV-mapping reads than that of the samples that did not receive this treatment (Table 1). This difference was especially pronounced when looking at the group of samples that underwent virion purification using a sucrose gradient, in which the samples that also had their ARV-cDNA amplified via R-SPA (S13–S16) presented a significantly higher number of ARV-mapping reads (*p* = 0.0485) than when no amplification was performed (S9–S12). Six samples (S1, S5, S9, S10, S11 and S12) presented <10% of mapped reads that translated to failed assemblies, incomplete genomes and low coverage (Table 1). The remainder of the samples showed an average coverage depth > 35X, with the highest average coverage depth above 950X for the sample treated with the Capto Core 700 resin and Benzonase. Surprisingly, a large average depth does not always correlate with obtaining complete genomes. The average ARV genome size is ~23,500 bps, and while most of the samples that were purified with Capto Core 700 produced contigs that covered at least 90% of the ARV genome, none of the samples enriched via the sucrose step gradient achieved more than 75% genome coverage.

These results provide evidence that the ARV-cDNA amplification step via R-SPA is essential to achieve a higher yield of ARV-mapping reads from the samples. Therefore, to continue narrowing down the best ARV-purification protocol for WGS, this experiment was repeated with two more plaque-purified ARV clinical isolates (strain ARV_94594 and ARV_126484).

### 3.2. Optimization of Quality Reads for ARV Genome Purification

**Figure 2:**
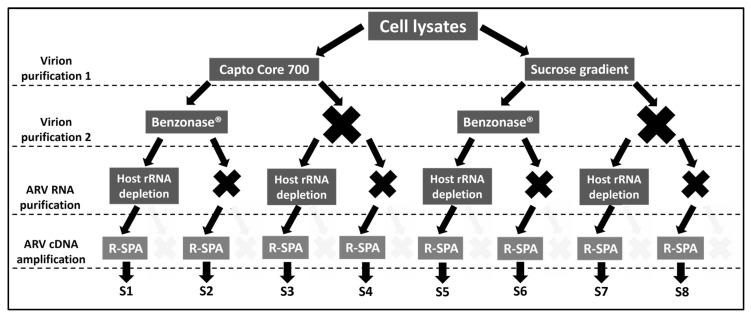
Schematic representation of experiment 2. Two ARV clinical isolates were expanded in LMH cells and purified using eight different combinations (S1–S8) of four ARV purification/amplification methods (left).

In a second experiment, the number of purification strategies to test was reduced from sixteen to eight because all the purified ARV genomes were subjected the ARV-cDNA amplification step (Figure 2). Eight purification strategies were tested in two different cell lysates infected with the strains ARV_94594 and ARV_126484 (Tables S2A and S3). Hence, the number of samples included in the second sequencing run was again sixteen.

The sixteen genomes sequenced resulted in a total of 541,754 (36,117 ± 8787 (Mean ± SEM)) raw reads, from which 443,425 (~82%) passed the quality filtering. Table 2 summarizes the average results obtained for the purification of ARV strain Alabama, ARV_94594 and ARV_126484. No significant differences (*p* = 0.0658) were found when comparing the number of raw reads and quality-filtered reads (*p* = 0.4182) obtained from the samples treated using the different purification methods. However, the proportion of reads mapping with the chicken genome (*p* = 0.0464) and with ARV significantly differed (*p* = 0.0402) between the different ARV purification treatments. As observed in the previous experiment, the administration of Benzonase or the host rRNA depletion treatments alone or in combination decreased the number of chicken genome-mapping reads, while increasing the number of ARV reads independently of the virion purification method used. Overall, the combination of Capto Core 700 resin (with or without the application of Benzonase) for virion purification and host rRNA depletion after total RNA extraction yielded a higher proportion of ARV-mapping reads in the samples (>80%, Table 2). Consequently, the samples subjected to this combination of purification treatments resulted in a smaller number of contigs after de novo assembly, with the majority of the contigs mapping the ARV reference genome S1133. Furthermore, the purification strategies in which a combination of Capto Core 700 resin and host rRNA depletion was used were the only ones with which complete genomes were consistently obtained (Table 2). Overall, the results of this experiment strongly suggest that the combination of Capto Core 700 resin and chicken rRNA depletion is superior to any other purification protocol tested herein, and we demonstrated that the addition of Benzonase for further ARV virion purification does not significantly increase (*p* = 0.2473) the number of reads mapped to the ARV genome after the resin treatment.

**Figure 3:**
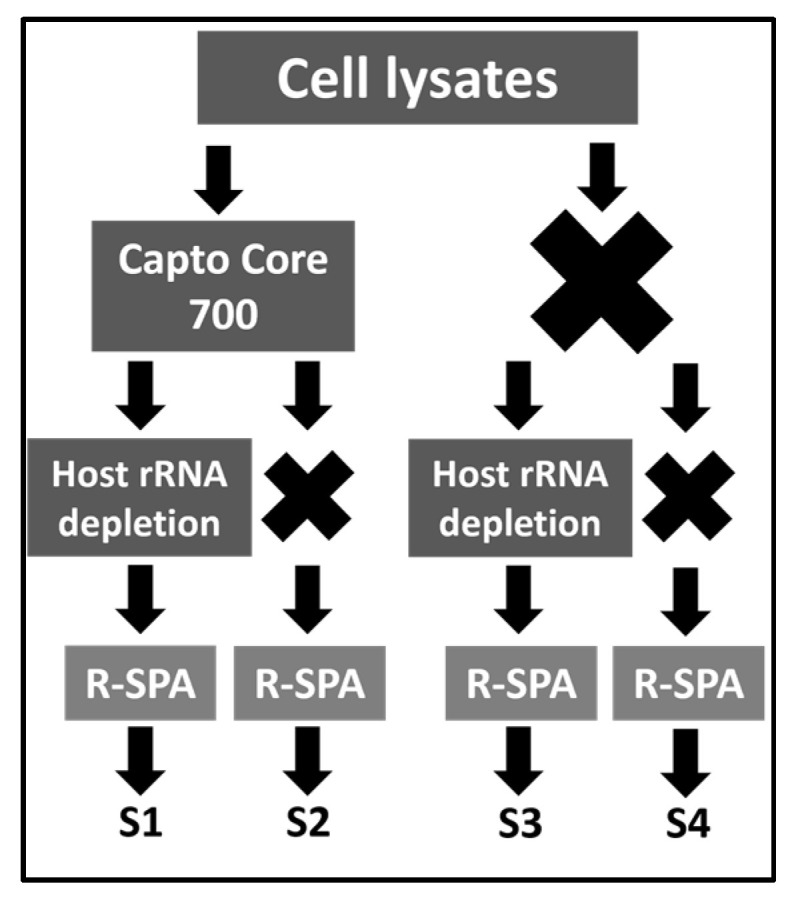
Schematic representation of experiment 3. The virions of three ARV clinical isolates and vaccine strain S1133 (n = 4 technical replicates) expanded in LMH cells were purified using four different combinations (S1–S4) of ARV purification/amplification methods (left).

The performance of the Capto Core 700 and chicken rRNA depletion combination method was tested in a final experiment that included the genome purification and amplification of three ARV clinical isolates (ARV_99846, ARV_106764 and ARV141045) without prior plaque-purification, and the ARV vaccine strain S1133 was a plaque-purified control. To solidify the findings described above, each of the purification steps had a negative control sample in which that particular step or combination of steps was not performed (Figure 3).

**Figure 4:**
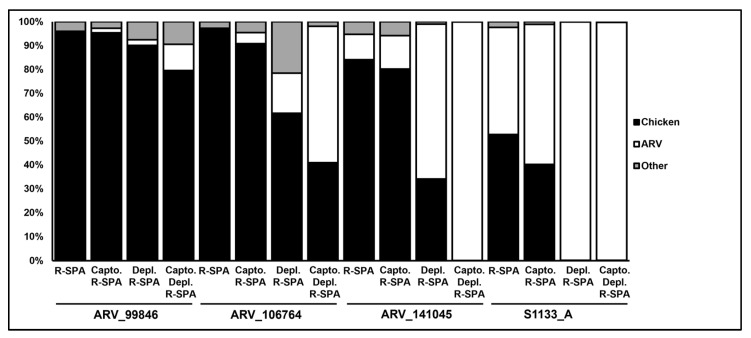
Percentage of quality-filtered reads found to map with chicken reference genome (black), ARV (white) or unmapped ones (“other”, gray). Reads were obtained from ARV clinical isolates, ARV_99846, ARV_106764 and ARV_141045, and vaccine strain S1133 that received R-SPA treatment only (R-SPA) or in combination with Capto Core 700 (Capto. R-SPA), host rRNA depletion (Depl. R-SPA) or both (Capto. Depl. R-SPA).

In total, sixteen samples were included in this new experiment (four previously untested ARV isolates under four purification conditions) that yielded a total of 365,924 raw reads (Table S2B), with 210,772 (57.6% of total raw reads) passing the quality filter (Table 3). There were no significant differences in the number of raw reads (*p* = 0.6452) and filtered reads (*p* = 0.6593) between the different purification treatments. The proportion of reads that mapped with ARV and the chicken genome did not significantly differ (*p* = 0.1766) between the treatments due to the high variability between the samples. However, the ‘Capto Core 700 and chicken rRNA depletion’ combination consistently outperformed the controls (Figure 4). The costs associated with the purification, amplification and sequencing of the ARV genome were calculated and compared (Table S4). The data generated in this study indicate that an average coverage of approximately 200X is required to have a minimum of 30X coverage for every segment of the virus. Under ideal conditions (all the reads on a sample belonging to ARV), 28,200 250-bp reads would be sufficient to cover the complete genome two hundred times over (Table S4A). When no ARV enrichment approaches are implemented and <0.1% of the reads of a sample are expected to map into the viral genome, ~2,820,000 250-bp reads (ARV and no-ARV) must be sequenced to obtain 200X sequencing depth of the ARV genome. With more reads required per sample, there is a decrease in the number of samples that can be included in a single sequencing run, and this increases the cost of sequencing per sample. When no ARV purification or enrichment is performed, the estimated cost of RNA extraction and sequencing is ~USD 450. This price decreases dramatically with the application of any of the purification strategies detailed herein, to between USD 104–USD 110 a sample (Table S4B). Although the combination of Capto Core 700 resin for ARV virion purification and R-SPA for ARV genome amplification appears to be the most affordable (USD 104), sufficient reads to create contigs that cover the whole ARV genome were only retrieved when a host rRNA depletion step was applied (Table 2 and Table 3). Adding this crucial step increased the cost of sample processing by a trivial amount (USD 2), for a total cost of USD 106/genome (Table S4B).

### 3.3. Short-Read Sequencing Can Detect a Mix of ARVs in a Clinical Isolate

The ARV genome purification and amplification steps had a bigger impact on the plaque purified vaccine strain ARV S1133 than they did on the clinical isolates. Indeed, the performance of the Capto Core 700 and host rRNA depletion treatments combination was below the expectation (>80% AVR-mapping reads based on the previous experiments described above) for two of the three clinical isolates, ARV_99846 and ARV_106764. The de novo assembly of the reads obtained from the purified and amplified genomes of ARV_141045 and S1133 yielded complete genome sequences (NCBI BioProject with accession number PRJNA993669) comprising a small number of contigs (~1 per ARV genome segment) with good coverage (>X50). On the other hand, the de novo assembly of ARV_99846 and ARV_106764 reads (from Capto Core 700-treated and host rRNA-depleted samples) produced an unexpectedly higher number of ARV and no-ARV contigs that did not form complete genome sequences (Table 3). Furthermore, the ARV_106764 sample treated with Capto Core 700 and host rRNA depletion showed an estimated genome length (48,244 bp) that doubled the average ARV genome size (~23,949 bp), indicating that the clinical isolate could be a mix of two ARV strains.

**Figure 5:**
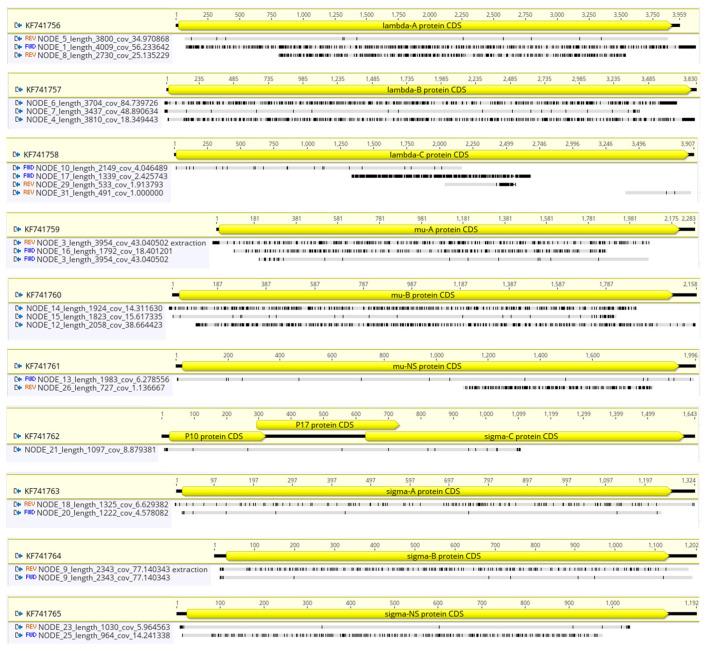
ARV_106764 assemblies (contigs NODE 1; 3–10; 12–18; 20; 21; 23; 25; 26; 29 and 31) mapped to ARV S1133 (NCBI accession number KF741756-65). S1133 genome annotations indicated with a yellow arrow. Numbers on top of the arrow show the length of the ARV genomic segment. Contig length (bp) and coverage are included on the contig’s name. REV indicates the reverse and complimentary sequence of the contig has been mapped. FWD (or not specified) indicates the forward sequence of the contig has been mapped. Grey areas in the alignment represent homology with the reference genome, while black areas show disagreement.

Twenty-three contigs generated by the de novo assembly of ARV_106764 after Capto Core 700 virion purification and host rRNA depletion where further investigated to determine if more than one ARV strain was present in the clinical isolate. The alignment of ARV_106764 contigs with the vaccine strain S1133 reference genome elucidated that all the ARV genomic segments, excepting S1 (NCBI accession no. KF741762), presented two or more ARV_106764 contigs sharing different homologies between them and the reference genome (Figure 5). Seventeen out of the twenty-three contigs covered >90% of the segment length, and twelve presented at a depth above 15X, suggesting that they are not sequencing artifacts. Interestingly, for every segment, one contig shared a high level of homology with the genome of S1133 (>98% homology), while the other contig(s) did not (<91% homology). Regardless, these results demonstrate that short-read DNA sequencing can be used to detect a mix of ARVs in a clinical isolate.

## 4. Discussion

This study aimed to determine the most efficient and cost-effective ARV genome enrichment strategy for WGS purposes. Most of the non-target reads produced via ARV-WGS come from the host (in our case, the chicken), which in some cases, reaches > 99% of the sequenced material [2]. Theoretically, there are three options to enrich the ARV genetic content in a sample before sequencing: (i) reducing the chicken genetic content, (ii) increasing the ARV genetic content, or (iii) a combination of both. A reduction of the chicken genetic content in a sample can be accomplished before extracting the viral genetic material (while the virions are still intact) via virion purification, or after, by depleting the chicken DNA/RNA present in the sample. We included these two approaches in this study. We compared the performance of ultracentrifugation in a sucrose density gradient (a commonly used technique for virion purification [1,23]) to that of a protocol developed by James et al. that uses Capto Core 700 resin to purify ARV virions [14]. Additionally, we tested a treatment with Benzonase after virion purification to further purify the intact virions before opening them for RNA extraction. Benzonase is an engineered endonuclease that degrades all forms of DNA and RNA, while having no proteolytic activity. Hence, we reasoned that Benzonase could help degrade residual host nucleic acids without impacting dsRNA protected within intact virions. The last purification step performed in this study to reduce the chicken genomic content was a host rRNA depletion treatment using a set of sixty-nine ~120 bp non-overlapping DNA probes covering the entire length of the chicken 18S and 28S ribosomal RNAs (rRNAs) and mitochondrial RNAs (mtRNAs) [15]. These probes hybridize with target chicken RNAs, forming RNA:DNA complexes that are degraded via subsequent treatments with RNaseH (endonuclease that catalyzes the cleavage of RNA bound to DNA) first and DNaseI (endonuclease that nonspecifically cleaves DNA) after. The amplification of the ARV genetic content in a sample can be conducted via PCR amplification of the viral genome before NGS [24,25] or via target capture NGS [26]. While we are unaware of an available target capture NGS scheme with ARV-specific probes, early this year, Chrzastek and collaborators reported a single primer amplification assay (R-SPA) that significantly improved the recovery of ARV mapping reads after sequencing [13]. This approach was evaluated in the experiments documented herein, alone and in combination with the other ARV purification methods mentioned above.

Our results show that Capto Core 700 by itself slightly, but consistently, increases the number of ARV-mapping reads compared to when not virion purification is applied. Additionally, we observed that the sucrose gradient by itself works better than Capto Core 700 by itself at purifying ARV virions, but it also seems to decrease the amount of ARV in the sample quite dramatically. Losing a virion or viral RNA is expected after every purification step, and generally, this small loss is compensated by a high purity. The application of Benzonase and host rRNA depletion increased the yield of ARV reads from virions purified via Capto Core 700, but not via ultracentrifugation in a sucrose gradient. Capto Core 700 resin is made of porous hollow beads with an inactive shell and a ligand-activated core that traps and holds molecules smaller than viruses (under 700 kilodaltons) [27]. Hence, small DNA/RNA fragments are expected to enter the core of the beads, bind to the internal ligands and be depleted from the sample. This indicates that the majority of the host contamination left after virion purification with Capto Core 700 are long nucleotide (DNA and RNA) sequences that are depleted with the subsequent enzymatic treatments (Benzonase and host rRNA depletion). We did not explore the application of Benzonase prior to virion purification with Capto Core 700, but this may improve the purification process even further. The further purification of virions isolated via ultracentrifugation in a sucrose gradient with host rRNA depletion seemed to negatively affect the ARV-read yield after NGS, and the application of Benzoanse does not improve the purity as drastically as it did for the Capto Core 700-treated samples in our experiments. Ultracentrifugation over a sucrose density gradient uses the high density (and therefore, lower buoyancy) of the viral particles, which travel across the gradient, to separate them from the host cell debris (membranes, organelle fragments and nucleic acids) that become trapped in between the sucrose layers [14,23]. We suspect that this technique, while purifying the virions, may reduce their numbers considerably more than the Capto Core 700 resin one can, and therefore, the subsequent purification steps (that always carry a minimal sample loss) more dramatically impact the outcome.

On the other hand, the amplification of ARV cDNA with R-SPA consistently improved the yield of ARV reads in the samples regardless of the purification method(s) used to remove the chicken genomic content. For this reason, only the strategies that included the R-SPA step were investigated in our subsequent experiments. The results obtained by applying only R-SPA to the samples (Figure 4, average of 14% ARV reads in a sample) were consistent with the results reported by Chrzastek et al. [13]. However, we observed that R-SPA seemed to work better for the vaccine strain S1133 (~45% ARV reads) than it did for the clinical isolates (<11% ARV reads). We first hypothesized that S1133 (vaccine strain and reference genome) was most probably among the group of strains used to design the R-SPA oligos, and therefore, we observed a better performance with this isolate. To test this hypothesis, we compared the sequence homology of R-SPA primer R8N [13] with the ARV whole genomes obtained in this study, and we observed that the 5′ end of the ARV fragments (where R8N aligns) is very conserved, and therefore, it is very unlikely that the observed differences in the R-SPA performance were caused by different primer affinities between the ARV strains. Another potential explanation resides in the fact that although all the isolates were expanded in LMH cells for the same period of time, S1133 is cell-culture-adapted, so it could be disproportionately expanded compared to the other clinical isolates, yielding a higher viral titer, and therefore, more viral genomes for PCR amplification to act upon. This second hypothesis could not be tested because the techniques for quantifying ARV titers (plaque forming units, PFU) and/or genomes (qPCR) are not developed or well standardized. Still, when combined with any other ARV purification method (at the virion level or after RNA extraction), R-SPA consistently improved the enrichment of ASV reads in each sample.

The combination of virion purification with Capto Core 700 (with or without Benzonase treatment) followed by host rRNA depletion and cDNA amplification was the most cost-effective approach and the only strategy that allowed the assembly of whole genomes. This was unexpected as the average genome coverage estimated with the ARV reads of the samples purified with this combination was comparable to the average genome coverage of the samples that went through other enrichment strategies, demonstrating that the latter one yielded enough ARV reads to produce complete genomes as well. The main difference between the samples from which we obtained complete genomes and the other samples is the level of host contamination present in the sample, indicating that high levels of non-ARV contamination (>50%) negatively impact the assembly of whole genomes. Additionally, the combination of Capto Core 700 host rRNA depletion and cDNA amplification allowed the detection of a mix of ARVs in a clinical isolate. However, the efficiency of the enrichment strategy was lower in the mixed samples (<57% ARV reads) compared to that of the isolates that only contained one ARV isolate (>82%).

## 5. Conclusions

The results obtained in this set of experiments that included three independent NGS runs and seven ARV isolates demonstrated that the combination of Capto Core 700 resin for virion purification, host rRNA depletion and ARV cDNA amplification is the most cost-effective strategy to enrich the ARV genomic content in a sample prior to NGS.

## Figures and Tables

**Table 1:** Summary of the WGS data generated from ARV Alabama treated with sixteen pre-sequencing purification strategies.

ID	Virion Purification Method	Benzonase Treatment	Host rRNA Depletion	R-SPA	Filtered Reads	Chicken- Mapping Filtered Reads	ARV-Mapping Filtered Reads	Total Contigs	ARV Contigs	Estimated Genome Coverage	Estimated Genome Length (bp)
S1	CaptoCore700	-	-	-	66,527	99.8%	0	0	0	0	0
S2	CaptoCore700	Benzonase	-	-	204,945	54.2%	45.1%	32	23	951	24,320
S3	CaptoCore700	-	Depletion	-	44,945	63.3%	39.0%	19	13	202	20,769
S4	CaptoCore700	Benzonase	Depletion	-	7879	43.0%	52.7%	59	30	36	26,963
S5	CaptoCore700	-	-	R-SPA	30,665	98.3%	0.1%	0	0	0	0
S6	CaptoCore700	Benzonase	-	R-SPA	22,516	32.8%	50.7%	12	10	157	21,566
S7	CaptoCore700	-	Depletion	R-SPA	12,482	32.3%	56.7%	14	11	87	22,381
S8	CaptoCore700	Benzonase	Depletion	R-SPA	17,978	30.6%	59.1%	20	14	130	23,638
S9	Sucrose	-	-	-	1082	85.8%	6.1%	0	0	0	0
S10	Sucrose	Benzonase	-	-	7419	61.3%	4.9%	76	15	10	10,064
S11	Sucrose	-	Depletion	-	1235	95.5%	0.8%	0	0	0	0
S12	Sucrose	Benzonase	Depletion	-	971	78.1%	1.6%	0	0	0	0
S13	Sucrose	-	-	R-SPA	81,379	75.6%	22.6%	24	17	671	12,081
S14	Sucrose	Benzonase	-	R-SPA	35,942	22.2%	77.6%	13	12	316	22,011
S15	Sucrose	-	Depletion	R-SPA	150,048	83.6%	16.2%	15	13	435	15,486
S16	Sucrose	Benzonase	Depletion	R-SPA	71,016	65.5%	34.4%	19	13	300	18,042

The samples that received ARV cDNA amplification with R-SPA are shown in grey. Genome coverage describes the number of unique reads that include a given nucleotide in the reconstructed sequence.

**Table 2:** Summary of the WGS data generated from three ARV isolates treated with eight pre-sequencing purification strategies.

Virion Purification Method	Benzonase Treatment	Host rRNA Depletion	R-SPA	Filtered Reads	% Chicken- Mapping Filtered Reads	% ARV- Mapping Filtered Reads	Total Contigs	ARV Contigs	Average Genome Coverage	Estimated Genome Length (bp)
CaptoCore700	-	-	R-SPA	19,664 ± 7907	98 ± 0.2	1.2 ± 0.5	5 ± 5	5 ± 5	3 ± 3	
CaptoCore700	Benzonase	-	R-SPA	37,309 ± 15,084	55 ± 18	26 ± 20	19 ± 5	10 ± 1	163 ± 5	
CaptoCore700	-	Depletion	R-SPA	15,913 ± 5216	11 ± 10	82 ± 13	13 ± 1	11 ± 1	135 ± 35	
CaptoCore700	Benzonase	Depletion	R-SPA	16,820 ± 5457	10 ± 10	86 ± 14	15 ± 3	13 ± 2	146 ± 37	
Sucrose	-	-	R-SPA	45,163 ± 20,557	55 ± 12	40 ± 10	17 ± 4	14 ± 2	352 ± 172	
Sucrose	Benzonase	-	R-SPA	46,464 ± 23,480	39 ± 28	57 ± 25	53 ± 38	30 ± 15	158 ± 80	
Sucrose	-	Depletion	R-SPA	87,287 ± 42,323	42 ± 24	53 ± 25	24 ± 15	15 ± 1	349 ± 167	
Sucrose	Benzonase	Depletion	R-SPA	28,528 ± 18,476	38 ± 20	62 ± 20	17 ± 1	13 ± 1	140 ± 79	

Numbers reflect the Mean ± SEM (n = 3) except for the complete genome column that shows the number of complete genomes obtained which each purification strategy. Genome coverage describes the number of unique reads that include a given nucleotide in the reconstructed sequence. The percentage of reads that map with chicken and ARV genomes is shown in grey.

**Table 3:** Summary of the WGS data generated from ARV isolates ARV_99846, ARV_106764, ARV141045 and S1133 treated with four pre-sequencing purification strategies.

ID	Virion Purification Method	Host rRNA Depletion	R-SPA	Total Filtered Reads	% Chicken- Mapping Filtered Reads	% ARV- Mapping Filtered Reads	Total Contigs	ARV Contigs	Average Genome Coverage	Estimated Genome Length (bp)
ARV_99846	-	-	R-SPA	17,573	96	0	0	0	0	0
	CaptoCore700	-	R-SPA	13,942	95	2	3	3	12	6637
	-	Depletion	R-SPA	12,056	90	2	18	10	12	8229
	CaptoCore700	Depletion	R-SPA	11,045	80	11	36	19	16	23,329
ARV_106764	-	-	R-SPA	5403	97	0	0	0	0	0
	CaptoCore700	-	R-SPA	14,616	91	5	6	5	20	10,214
	-	Depletion	R-SPA	7562	62	17	26	23	13	29,403
	CaptoCore700	Depletion	R-SPA	34,643	41	57	33	23	130	48,244
ARV_141045	-	-	R-SPA	7743	84	11	11	11	12	17,087
	CaptoCore700	-	R-SPA	30,754	80	14	12	11	66	21,552
	-	Depletion	R-SPA	13,036	35	66	11	10	109	23,135
	CaptoCore700	Depletion	R-SPA	11,408	0	100	11	11	115	22,870
S1133	-	-	R-SPA	4731	53	45	8	8	35	16,186
	CaptoCore700	-	R-SPA	5302	40	59	9	9	47	19,113
	-	Depletion	R-SPA	16,006	0	100	10	10	152	23,499
	CaptoCore700	Depletion	R-SPA	4952	0	100	10	10	50	22,681

Genome coverage describes the number of unique reads that include a given nucleotide in the reconstructed sequence. The number of reads that map with chicken and ARV genomes are shown in grey.

## Data Availability

The complete genomes of the ARVs sequenced in this study were deposited in NCBI BioProject under accession number PRJNA933177.

## Supplementary Materials

The following supporting information can be downloaded at: https://www.mdpi.com/article/10.3390/v15091938/s1, Table S1: List of ARV isolates used in this study; Table S2. Raw reads obtained per sample on each sequencing run. Table S3. Summary of the WGS data generated from ARV isolates ARV_Alabama in sequencing run 1 and, ARV_94594 and ARV_126484 in sequencing run 2. Table S4: Costs associated with the implementation of ARV genome purification and amplification steps before WGS.
