# Supplementary material for: Optimizing the Conditions for Whole-Genome Sequencing of Avian Reoviruses
Source: Viruses. 2023 Sep 16;15(9):1938. doi: 10.3390/v15091938 (PMC10536876; doi:10.3390/v15091938)
Supplement: Supplementary file 1 [file viruses-15-01938-s001.zip › viruses-2531547 Supplementary_Material_reviewed.docx.pdf]

viruses MDPI

Table S1. ARV isolates used in this study.

ID	Host	Pathology	Collection year	Location	NCBI Biosample no.
Alabama	Chicken	Mild lymphocytic myocarditis and moderate hepatic sinusoidal congestion	2021	AL:USA	SAMN36407456
ARV_94594	Chicken	Tenosynovitis	2012	AL:USA	SAMN36407457
ARV_126484	Chicken	Tenosynovitis	2018	IA:USA	SAMN36407458
ARV_99846	Chicken	Tenosynovitis	2013	NC:USA	SAMN36407459
ARV_106764	Chicken	Tenosynovitis	2014	IA:USA	SAMN36407460
ARV141045	Chicken	Tenosynovitis	2021	AL:USA	SAMN36407461
S1133	Vaccine	-			SAMN36407462

Table S2. Raw reads obtained per sample on each sequencing run.

A) Sequencing runs 1 and 2

Virion Purification Method	Benzonase Treatment	Host rRNA Depletion	R-SPA	#Exp1 ARV_Alabama	#Exp2 ARV_94594	#Exp2 ARV_126484
CaptoCore700	-	-	-	76,080	-	-
CaptoCore700	Benzonase	-	-	253,978	-	-
CaptoCore700	-	Depletion	-	64,268	-	-
CaptoCore700	Benzonase	Depletion	-	18,412	-	-
CaptoCore700	-	-	R-SPA	45,206	28,624	8,388
CaptoCore700	Benzonase	-	R-SPA	37,816	60,256	-
CaptoCore700	-	Depletion	R-SPA	34,178	33,338	16,700
CaptoCore700	Benzonase	Depletion	R-SPA	27,498	27,912	14,954
sSucrose Gradient	-	-	-	17,460	-	-
Sucrose Gradient	Benzonase	-	-	20,274	-	-
Sucrose Gradient	-	Depletion	-	5,110	-	-
Sucrose Gradient	Benzonase	Depletion	-	6,920	-	-
Sucrose Gradient	-	-	R-SPA	98,618	50,016	23,972
Sucrose Gradient	Benzonase	-	R-SPA	79,140	17,332	105,092
Sucrose Gradient	-	Depletion	R-SPA	194,348	13,882	118,070
Sucrose Gradient	Benzonase	Depletion	R-SPA	87,704	6,918	16,300

B) Sequencing run 3

Virion Purification Method	Host rRNA Depletion	R-SPA	Raw reads ARV_99846	Raw reads ARV_106764	Raw reads ARV_141045	Raw reads S1133
-	-	R-SPA	30,674	7,018	15,550	8,870
CaptoCore700	-	R-SPA	22,520	22,756	61,896	11,062
-	Depletion	R-SPA	16,762	14,830	22,702	25,746
CaptoCore700	Depletion	R-SPA	18,180	58,940	16,734	11,684

Table S3. Summary of the WGS data generated from ARV isolates ARV_Alabama in sequencing run 1 and, ARV_94594 and ARV_126484 in sequencing run 2.

Virus ID	Virion Purification Method	Benzonase Treatment	Host rRNA Depletion	R-SPA	Filtered reads	%Chicken-mapping Filtered Reads	%ARV-mapping Filtered Reads	Total Contigs	ARV Contigs	Estimated Genome Coverage	Estimated Genome Length (bp)
ARV_94594	s	-	-	R-SPA	24,518	98	2	15	14	8	15,510
	CaptoCore700	Benzonase	-	R-SPA	52,093	77	25	25	11	169	18,754
	CaptoCore700	-	Depletion	R-SPA	21,951	2	75	14	12	204	23,251
	CaptoCore700	Benzonase	Depletion	R-SPA	21,349	0	73	9	9	217	23,134
	Sucrose gradient	-	-	R-SPA	40,499	33	58	16	14	307	22,234
	Sucrose gradient	Benzonase	-	R-SPA	11,788	2	71	18	17	94	23,236
	Sucrose gradient	-	Depletion	R-SPA	5,972	43	50	32	15	36	20,427
	Sucrose gradient	Benzonase	Depletion	R-SPA	4,683	48	47	16	11	28	19,988
ARV_126484	CaptoCore700	-	-	R-SPA	6,343	98	2	-	-	-	-
	CaptoCore700	Benzonase	-	R-SPA	-	-	-	-	-	-	-
	CaptoCore700	-	Depletion	R-SPA	13,247	0	81	10	10	115	23,241
	CaptoCore700	Benzonase	Depletion	R-SPA	11,014	0	77	15	15	92	23,038
	Sucrose gradient	-	-	R-SPA	14,022	57	50	11	11	79	22,845
	Sucrose gradient	Benzonase	-	R-SPA	91,542	94	11	128	60	64	21,385
	Sucrose gradient	-	Depletion	R-SPA	112,316	0	60	26	16	541	18,752
	Sucrose gradient	Benzonase	Depletion	R-SPA	12,088	0	78	16	16	98	23,172
ARV_Alabama	CaptoCore700	-	-	R-SPA	30,665	98	0.1	0	0	0	0
	CaptoCore700	Benzonase	-	R-SPA	22,516	33	51	12	10	157	21,566
	CaptoCore700	-	Depletion	R-SPA	12,482	32	57	14	11	87	22,381
	CaptoCore700	Benzonase	Depletion	R-SPA	17,978	31	59	20	14	130	23,638
	Sucrose gradient	-	-	R-SPA	81,379	76	23	24	17	671	12,081
	Sucrose gradient	Benzonase	-	R-SPA	35,942	22	77	13	12	316	22,011
	Sucrose gradient	-	Depletion	R-SPA	150,048	84	16	15	13	435	15,486
	Sucrose gradient	Benzonase	Depletion	R-SPA	71,016	66	34	19	13	300	18,042

viruses MDPI

Table S4. ARV read enrichment (A) and cost associated (B) with the implementation of each strategy A)

	**No Purification/ Enrichment**	**R-SPA**	**Capto. R-SPA**	**Depl. R-SPA**	**Capto. Depl. R-SPA**	**Ideal Conditions**
Proportion of ARV reads in library	0.1%	17%	24%	52%	77%	100%
Reads for X200 genome coverage	2,820,000	165,882	117,500	54,231	36,623	28,200
Libraries per cartridge	1	12	17	37	55	71

B)

	**No Purification/ Enrichment**	**R-SPA**	**Capto. R-SPA**	**Depl. R-SPA**	**Capto. Depl. R-SPA**
Capto Core 700	-	-	3	-	3
RNA extraction	7	7	7	7	7
Host-rRNA depletion	-	-	-	18	18
R-SPA	-	25	25	25	25
Gnomic library prep	45	45	45	45	45
Cartridge price per sample	400	33	24	11	7
Price per genome	452	110	104	106	106
